# The influence of age, delay of repair, and tendon involvement in acute rotator cuff tears: Structural and clinical outcomes after repair of 42 shoulders

**DOI:** 10.3109/17453674.2011.566144

**Published:** 2011-04-05

**Authors:** Hanna C Björnsson, Rolf Norlin, Kajsa Johansson, Lars E Adolfsson

**Affiliations:** ^1^Department of Clinical and Experimental Medicine, Division of Inflammation Medicine, Orthopaedics and Sports Medicine, Linköping University and University Hospital, Linköping; ^2^Department of Orthopaedics, örebro University Hospital, örebro; ^3^Department of Medical and Health Sciences, Division of Physiotherapy, Linköping University, Linköping, Sweden

## Abstract

**Background and purpose:**

Few authors have considered the outcome after acute traumatic rotator cuff tears in previously asymptomatic patients. We investigated whether delay of surgery, age at repair, and the number of cuff tendons involved affect the structural and clinical outcome.

**Patients and methods:**

42 patients with pseudoparalysis after trauma and no previous history of shoulder symptoms were included. A full-thickness tear in at least 1 of the rotator cuff tendons was diagnosed in all patients. Mean time to surgery was 38 (6–91) days. Follow-up at a mean of 39 (12–108) months after surgery included ultrasound, plain radiographs, Constant-Murley score, DASH score, and western Ontario rotator cuff (WORC) score.

**Results:**

At follow-up, 4 patients had a full-thickness tear and 9 had a partial-thickness tear in the repaired shoulder. No correlation between the structural or clinical outcome and the time to repair within 3 months was found. The patients with a tendon defect at follow-up had a statistically significantly lower Constant-Murley score and WORC index in the injured shoulder and were significantly older than those with intact tendons. The outcomes were similar irrespective of the number of tendons repaired.

**Interpretation:**

A delay of 3 months to repair had no effect on outcome. The patients with cuff defects at follow-up were older and they had a worse clinical outcome. Multi-tendon injury did not generate worse outcomes than single-tendon tears at follow-up.

“Codman's trauma theory” postulated that trauma may rupture healthy rotator cuff tendons but that the rupture most often occurs in cases where aged tendons are weakened by overuse or degeneration (Fukuda 2000, Sorensen et al. 2007). Still, cuff ruptures with sudden pseudoparalysis occurring after trauma are usually considered acute, and immediate repair has been recommended (Bassett and Cofield 1983, Lahteenmaki and Lawrence 2007, Petersen and Murphy 2011). Almost all previous studies concerning rotator cuff repair have included patients with both acute and chronic degenerative tears. In the clinical setting, it is difficult to distinguish an acute tear from a degenerative one with acute symptoms after trauma (Bassett and Cofield 1983, Lahteenmaki and Lawrence 2007, Sorensen et al. 2007, Petersen and Murphy 2011). Since there have been few studies, with few participants and including only acute traumatic tears, little is known about the result after repair in this group of patients (Bassett and Cofield 1983, Lahteenmaki and Lawrence 2007, Petersen and Murphy 2011).

The Swedish national guidelines state that acute full-thickness tears with pseudoparalysis after trauma in previously asymptomatic patients should be repaired within 3 weeks (Swedish National Musculoskeletal Competence Centre 2006). There is little support for this guideline in the literature. To our knowledge, only a few previous studies have examined the effect of time to surgery after acute traumatic tear. Bassett and Cofield (1983) found that early repair within 3 weeks resulted in better shoulder function but Petersen and Murphy (2011) stated that the clinical outcome is not affected by a surgical delay of 4 months. As stated by Codman and others (Fukuda 2000, Sorensen et al. 2007, Perry et al. 2009, Duquin et al. 2010), most traumatic cuff tears occur in aged tendons. However, the influence of age on the results after acute cuff repair has not been studied in detail.

The number of cuff tendons injured has been reported to affect the structural and clinical outcomes in chronic tears, but as far as we know the same correlations have not been reported in patients with acute tears (Jost et al. 2006, Zingg et al. 2007, Nho et al. 2009a, Oh et al. 2009, 2010). Structural defects following repair of chronic tears have been reported to range between 13% and 94% (Fuchs et al. 2006, Jost et al. 2006, Zingg et al. 2007, Oh et al. 2009). To our knowledge, there have been no reports regarding maintenance of tendon integrity following repair of acute tears.

We investigated whether the structural and clinical outcomes after surgical repair of an acute rotator cuff tear in a previously asymptomatic patient are influenced by delay in repair, age at repair, and the extent of the initial cuff injury.

## Patients and methods

A retrospective review of our computerized database allowed us to identify 53 patients repaired with rotator cuff suture at the Department of Orthopaedics, Linköping University Hospital, between May 2004 and May 2009 due to an acute traumatic cuff tear. 42 patients who fulfilled the inclusion criteria (mean age at injury 59 (38–79) years, 32 men), were willing to participate in a follow-up evaluation and gave their written and oral informed consent (Table 1). The study was approved by the local ethics committee (Dnr. M128-09).

**Table 1.** Description of the study population

	All patients Mean (SD)	Intact group Mean (SD)	Defect group Mean (SD)	Mean difference [Intact – Defect]	p-value **^a^**
Days from trauma to surgical repair	38 (22)	39 (22)	38 (23)	0.6 (–14 to 15)	0.9
Months to follow-up from surgical repair	37 (23)	41 (23)	34 (24)	7.2 (–8.4 to 23)	0.4
Age at follow-up	62 (12)	60 (12)	68 (11)	–7.8 (–15 to –0.2)	0.05
% Women	24	24	23		1

**^a^** Independent Student's t-test comparing patients with no cuff defect (intact group) and patients with a cuff defect (defect group, including partial- and full-thickness tears).

The inclusion criteria were: trauma to the shoulder, sudden onset of symptoms, asymptomatic shoulder before trauma, pseudoparalysis, full-thickness rotator cuff tear of at least 1 tendon with an acute appearance when sutured, and no signs off previous cuff tearing or other cuff pathology. Patients with previous or gradual onset of symptoms in the injured shoulder, partial cuff tear, or displaced fracture were excluded. The acute cuff tears were diagnosed with MRI or ultrasound preoperatively, and were verified at surgery. Pseudoparalysis was defined as less than 45 degrees of motion in both active forward flexion and abduction. Due to the trauma, the rotator cuff tear was combined with other injuries in 19 of the patients. 2 patients had a non-displaced fracture of the greater tuberosity. The other 17 patients had a glenohumeral dislocation and, of these, 4 had a transient minor axillary nerve injury, 1 had a minor glenoid fracture, and 1 had a non-displaced fracture of the greater tuberosity after the dislocation. None of the associated injuries required additional surgery. In 41 patients, the full-thickness tear involved the supraspinatus tendon alone or in combination with other tendons and 1 patient had an isolated subscapularis tear (Table 2).

**Table 2.** Distribution of the peroperative acute full-thickness rotator cuff tear(s) in the patients in the study (n = 42)

Tendon tears	No. of patients
Isolated supraspinatus	14
Isolated subscapularis	1
Subtotal: single tendon tears	15
Supraspinatus, subscapularis	8
Supraspinatus, Infraspinatusi	7
Supraspinatus, subscapularis, infraspinatus	8
Supraspinatus, infraspinatus, teres minor	3
Supraspinatus, subscapularis, infraspinatus, teres minor	1
Subtotal: multiple tendon tears	27
Total	42

### Repair and postoperative rehabilitation

Randomly, and due to a timetabled service, 1 of 3 experienced shoulder surgeons performed the surgical procedure. Repair was performed with open technique using osteosutures and suture anchors in all patients, except for 1 arthroscopic procedure. The surgical approach was deltoid-split in 29 cases and deltopectoral in 12 cases. All preoperatively diagnosed full-thickness tears could be repaired. Open or arthroscopic subacromial decompression and bursectomy were performed in 35 patients.

Postoperatively, the patients used a shoulder immobilizer for 4 to 5 weeks. Rehabilitation was tutored by a physical therapist, beginning with pendulum and passive range-of-motion exercises. After the first 5 weeks, active range of motion was allowed and from 7–8 weeks strengthening exercises began with progress according to the individual patient involved. In cases with multi-tendon tears, including subscapularis, the shoulder was immobilized for 5 weeks and then similar exercises were started—but the strengthening exercises were delayed until week 9–10. Time from trauma to repair was on average 38 (6–91) days (Table 1); 11 patients were repaired within 3 weeks, 13 others within 3–6 weeks, and the remaining 18 within 6–12 weeks. Time from trauma to repair was due to delay in patients' referrals.

### Anatomical and clinical evaluation at follow-up

The mean length of follow-up was 39 (12–108) months (Table 1). All patients were examined with bilateral ultrasound at the Department of Radiology, Linköping University Hospital. An experienced radiologist and an orthopedic specialist interpreted the ultrasound independently of each other according to a standard protocol. The equipment used was a Siemens Acuson Sequoia 512 ultrasound machine with a variable 8–10 MHz linear array-transducer. The rotator cuff was evaluated in 2 planes with standardized positions and motions. The tendons were assessed as being intact, having a partial-thickness tear, or having a full-thickness tear. Partial-thickness tear was defined as a localized absence of the tendon, seen in 2 orthogonal imaging planes in less than the full-thickness of the tendon. Full-thickness tear was defined as no visualization of the tendon throughout the whole thickness. Thinner tendons but with full continuity were defined as being intact. Additionally, a radiographic examination of the glenohumeral and the acromio-clavicular joint bilaterally was performed in all but 1 patient, who declined the examination. An experienced radiologist and 2 shoulder specialists (HB and LA) assessed the radiographic findings independently of each other according to a protocol, including signs of osteoarthritis in the glenohumeral and acromio-clavicular joint, proximal humeral migration, and signs of subacromial degeneration (sclerosis, cysts, and spur formations). There was complete agreement between observers regarding classification of ultrasound and radiographic findings.

The follow-up clinical assessments were performed by 1 orthopedic specialist who was not involved in any of the formerly surgical procedures (HB), using a standardized interview and a per-protocol physical examination. Outcome measures used were the Constant-Murley score (Constant and Murley 1987), the DASH (disabilities of arm, shoulder, and hand) questionnaire (Atroshi et al. 2000) and the western Ontario rotator cuff (WORC) index (Kirkley et al. 2003). 2 patients did not complete the DASH questionnaire or the WORC index. To assess the possible influence of age, the total study sample was divided into patients who were older and younger than 65 years at follow-up. This age cut-off was chosen for comparison with previous published data using 65 years as a limit (Fehringer et al. 2008, Charousset et al. 2010).

### Statistics

The patients were divided into 2 groups at follow-up: those with a cuff defect (defect group) and those with intact tendons (intact group). Student's t-test and ANCOVA, with age as a covariate, were used for comparison of means between independent groups. Proportional differences between groups were analyzed using Pearson's Chi-square test or Fisher's exact probability test. All p-values < 0.05 were considered statistically significant and in this paper the wording “significant” or “significantly” always refers to statistical significance. SPSS statistical software version 17.0 was used for all analyses.

## Results

### Postoperative complications and additional surgery

3 patients had postoperative complications. 1 patient had a wound infection and 2 patients developed complex regional pain syndrome. During the time from cuff repair until the follow-up assessment, 5 patients developed impingement symptoms and were reoperated with arthroscopic subacromial decompression. In 4 of these 5 patients, subacromial decompression was not performed at the initial procedure for repair of the cuff. In the contralateral shoulder, 1 patient was operated with arthroscopic decompression during the follow-up time because of impingement symptoms. None of the patients included had sustained an additional trauma in any of their shoulders during the time from repair to follow-up.

### Ultrasound and radiographic results

Ultrasound examination of the operated shoulder identified 4 patients with a full-thickness tear and 9 with a partial-thickness tear. In the non-traumatised contralateral shoulder, 11 patients were found to have a full-thickness tear and 2 were found to have a partial-thickness tear (Table 3). The 4 patients with a full-thickness tear in the repaired shoulder also had a full-thickness tear in the contralateral shoulder. Osteoarthritis was identified in 2 patients, and in 1 of these patients it was bilateral (Table 3). Both of these patients also had full-thickness tears bilaterally at follow-up. Radiographic findings of subacromial degenerative changes were noted in the repaired shoulder in 2 patients and in the contralateral shoulder in 19 patients (Table 3).

**Table 3.** Structural evaluation at follow-up (ultrasound and radiographic examination)

	Repaired shoulder (n)	Contralateral shoulder (n)
Ultrasound		
Intact rotator cuff	29	29
Partial-thickness tear	9	2
Full-thickness tear	4	11
Radiography		
Osteoarthritis	2	1
Proximal humeral migration	4	5
Signs of subacromial degeneration	2	19

### Clinical results

At follow-up, patients with a cuff defect (defect group) were significantly older—mean age 68 (41–81) years—than the patients with no identifiable defect (intact group), who had a mean age of 60 (42–83) years (Table 1). However, when we divided the entire study sample into those who were older and those who were younger than 65 years at follow-up, no significant differences in clinical scores or in structural outcome could be found.

Mean time to repair was 38 (6–88) days in the defect group and 39 (8–91) days in the intact group (Table 1). No significant differences in Constant-Murley score, DASH score, or WORC index were found between the groups, irrespective of whether the repair had been performed within 3 weeks, within 6 weeks, or within 12 weeks. There was no significant relationship between the number of tendons involved at the primary injury and the clinical or structural outcomes. At follow-up, the patients with a cuff defect in the previously repaired shoulder had a significantly lower Constant-Murley score and WORC index in the repaired shoulder than the patients with intact tendons. The difference in DASH score between the two groups was not significant (Table 4). There was a significant difference in Constant-Murley score between the repaired shoulder and the contralateral shoulder in the defect group, but no significant difference between shoulders was found in the intact group.

**Table 4.** Clinical evaluation at follow-up

	All patients (n = 42) Mean (SD)	Intact group (n = 29) Mean (SD)	Defect group (n = 13) Mean (SD)	Mean difference (95% CI) [Intact – Defect]	Age-adjusted mean difference (95% CI) [Intact – Defect]	p-value **^a^**
Constant-Murley score						
repaired shoulder	67 (22)	73 (21)	55 (20)	17 (3 to 31)	16 (1 to 31)	0.04
contra- lateral shoulder	83 (18)	87 (14)	73 (22)	14 (0.2 to 28)	10 (–1 to 21)	0.07
WORC (%) **^b^**	75 (22)	79 (20)	65 (23)	14 (–2 to 30)	16 (1 to 32)	0.04
DASH **^b^**	22 (21)	17 (21)	31 (19)	–14 (–28 to 0.4)	–15 (–30 to 0.1)	0.05

**^a^** ANCOVA with age as a covariate, comparing patients with no cuff defect (intact group) and patients with cuff defect (defect group, including partial- and full-thickness tears).

**^b^** Missing values (n = 2).

## Discussion

Patients with sudden onset of pseudoparalysis due to an acute rotator cuff tear should, according to the Swedish guideline, be repaired within 3 weeks (Swedish National Musculoskeletal Competence Centre 2006). This guideline originates from a few studies based on clinical evaluation that suggested that early repair was beneficial (Bassett and Cofield 1983, Lahteenmaki and Lawrence 2007, Petersen and Murphy 2011). One of our aims was to assess whether patients repaired later have worse clinical and structural outcomes. In contrast to the findings of Bassett and Cofield (1983), we found that there were no statistically significant differences in any of the outcomes related to whether the repair had been performed within 3, 6, or 12 weeks of the trauma. Our results suggest that patients with a delayed repair, within a 3-month limit, may achieve the same satisfactory level of shoulder function as those with early repair. This is supported by the recently published study by Petersen and Murphy (2011) and by a study on rabbits that found an equal degree of surgical success if supraspinatus repair was performed immediately or up to 12 weeks after the tearing (Koike et al. 2006).

When the total study sample was divided into patients who were older or younger than 65 years, no significant differences were found in any of the clinical outcomes. Age should not be the only variable to consider in the clinical decision of whether or not to perform surgery (Oh et al. 2010). Based on our findings, age appears to affect the integrity of the cuff repair but no definite age limit can be defined. Similar findings have been presented in mid- to long-term follow-up studies involving both acute and chronic tears (Harryman et al. 1991, Adolfsson and Lysholm 1993, Zingg et al. 2007, Nho et al. 2009b, Oh et al. 2009, 2010).

Our results do not appear to support the idea that more than 1 full-thickness cuff tear results in worse mechanical properties and worse shoulder function after repair (Perry et al. 2009); nor do our findings support the view that patients with multiple tendon tears are initially predisposed to an increased risk of future structural defects than are patients with single tendon tears (Nho et al. 2009b, Oh et al. 2010). The clinical and structural outcomes were similar regardless of the number of initially injured tendons.

At follow-up, 4 of the 42 patients were found to have a full-thickness cuff defect in the operated shoulder, and 9 had a partial-thickness defect. All these defects were located in the previously repaired tendons, and may represent re-tearing, or tears that never fully healed. Interestingly, in addition to the 4 patients with bilateral full-thickness cuff defects, 7 other patients were identified as having a full-thickness cuff defect in the contralateral non-traumatized shoulder. This might suggest that the group of patients with a rotator cuff defect at follow-up has an ongoing degenerative process in both shoulders. A high prevalence of bilateral lesions has been reported in patients with chronic tears when evaluated 2 years after repair (Harryman et al. 1991). An increasing prevalence of asymptomatic tears with advancing age is well described, and despite the effort to include only acute tears in this study, some of our patients may have had cuff degeneration or even an asymptomatic rotator cuff tear in any shoulder before the trauma (Milgrom et al. 1995, Yamaguchi et al. 2001, Sorensen et al. 2007). Even though this study included only patients who were asymptomatic before the trauma and all sutured tears had an acute appearance at surgery, there is no way of ascertaining whether degenerative changes were already present. Given the fact that the incidence of cuff tears increases with age, and that they hardly ever appear before middle age, it is even likely that there is no such thing as an acute cuff tear without some previous tendon degeneration.

Subacromial decompression was performed in 35 of 42 of the patients. It might be speculated that the cuff-repaired shoulders with fewer full-thickness tears at follow-up than on the contralateral side (Table 3), could be explained by the subacromial decompression. The protective effect of this procedure on the rotator cuff has been suggested in previous studies (Adolfsson and Lysholm 1993, Chin et al. 2007, Bjornsson et al. 2010). 6 patients were operated with arthroscopic subacromial decompression in either of the shoulders during the follow-up period, which might have affected the clinical outcome. Of particular interest is the fact that in 4 of the 7 patients who did not have this procedure done as part of the cuff repair procedure, the decompression had to be performed at a later stage. In contrast, only 1 of the 35 patients who underwent simultaneous cuff suture and decompression developed impingement symptoms during the follow-up period. This suggests that subacromial decompression should be an integrated part of the cuff repair procedure.

When considering only chronic tears, divergent results concerning correlations between recurrent structural defects and the clinical outcomes such as shoulder function and pain have been reported (Harryman et al. 1991, Nho et al. 2009b, Oh et al. 2009, Zingg et al. 2007). The current trend is a better functional outcome with an intact repair at follow-up (Perry et al. 2009). This trend is supported by our study of acute lesions, with significantly better Constant-Murley score and WORC index in the intact group (Table 4).

The choice of ultrasound examination to investigate the structural condition of the rotator cuff has many advantages. It is highly accurate in evaluating the integrity of the rotator cuff after cuff repair, with a sensitivity of 100% and a specificity of 85%; also, the images are not distorted by the suture anchors (like MRI) and the evaluation is harmless regarding ionising radiation (Harryman et al. 1991, Teefey et al. 2004, Sorensen et al. 2007, Nho et al. 2009a, Oh et al. 2009).

The number of participants in our study limited the possibility of a complete multivariate analysis, since it gave too few subjects in the relevant subgroups. Our statistically significant differences have rather wide confidence intervals, which may affect the generalization of our results. It could be argued that the time to follow-up was short for some patients (range 12–108 months). However, Nho et al. (2009a) reported that all patients with an intact tendon after 1 year remained intact at the 2-year follow-up, indicating that longer follow-up would not make any difference.

A strength of our study is the structural follow-up of the rotator cuff tendons with both radiographic and ultrasound examination bilaterally, which made it possible to correlate the structural findings in both shoulders to the surgical procedures performed. Furthermore, both the ultrasound and radiographic assessments at follow-up were validated, since they were performed by 2–3 independent investigators who were in agreement about the findings. As far as we know, this is largest study with imaging techniques to assess the structural integrity at mid-term follow-up after repair of acute rotator cuff tears.

In conclusion, acute traumatic tears of the rotator cuff in previously healthy shoulders can be repaired with an open technique, at least up to 3 months after the injury. Patients with a rotator cuff defect at follow-up were significantly older and the defect resulted in significantly worse Constant-Murley score and WORC index in the repaired shoulder than in patients with an intact cuff. Patients with acute multiple tendon tears did not have a worse clinical or structural outcome at follow-up than patients with only 1 tendon tear.
